# Tetanus Toxin *cis*-Loop Contributes to Light-Chain Translocation

**DOI:** 10.1128/mSphere.00244-20

**Published:** 2020-05-06

**Authors:** Madison Zuverink, Matthew Bluma, Joseph T. Barbieri

**Affiliations:** a Microbiology and Immunology, Medical College of Wisconsin, Milwaukee, Wisconsin, USA; University of Maryland Medical Center

**Keywords:** *Clostridium*, cell biology, exotoxins, protein translocation, tetanus, toxins

## Abstract

The clostridial neurotoxins (CNTs) comprise tetanus toxin (TT) and botulinum neurotoxin (BoNT [BT]) serotypes (A to G and X) and several recently identified CNT-like proteins, including BT/En and the mosquito BoNT-like toxin Pmp1. CNTs are produced as single proteins cleaved to a light chain (LC) and a heavy chain (HC) connected by an interchain disulfide bond. LC is a zinc metalloprotease (cleaving soluble N-ethylmaleimide-sensitive factor attachment protein receptors [SNAREs]), while HC contains an N-terminal translocation domain (HCN) and a C-terminal receptor binding domain (HCC). HCN-mediated LC translocation is the least understood function of CNT action. Here, β-lactamase (βlac) was used as a reporter in discovery-based live-cell assays to characterize TT-mediated LC translocation. Directed mutagenesis identified a role for a charged loop (^767^DKE^769^) connecting α15 and α16 (*cis*-loop) within HCN in LC translocation; aliphatic substitution inhibited LC translocation but not other toxin functions such as cell binding, intracellular trafficking, or HCN-mediated pore formation. K^768^ was conserved among the CNTs. In molecular simulations of the HCN with a membrane, the *cis*-loop did not bind with the cell membrane. Taken together, the results of these studies implicate the *cis*-loop in LC translocation, independently of pore formation.

**IMPORTANCE** How protein toxins translocate their catalytic domain across a cell membrane is the least understood step in toxin action. This study utilized a reporter, β-lactamase, that was genetically fused to full-length, nontoxic tetanus toxin (βlac-TT) in discovery-based live-cell assays to study LC translocation. Directed mutagenesis identified a role for K^768^ in LC translocation. K^768^ was located between α15 and α16 (termed the *cis*-loop). Cellular assays showed that K^768^ did not interfere with other toxin functions, including cell binding, intracellular trafficking, and pore formation. The equivalent K^768^ is conserved among the clostridial neurotoxin family of proteins as a conserved structural motif. The *cis*-loop appears to contribute to LC translocation.

## Abstract (precis)

How protein toxins translocate their catalytic domain across a cell membrane is the least understood step in toxin action. This study utilized a reporter, β-lactamase, that was genetically fused to full-length, nontoxic tetanus toxin (βlac-TT) in discovery-based live-cell assays to study LC translocation. Directed mutagenesis identified a role for K^768^ in LC translocation. K^768^ was located between α15 and α16 (termed the *cis*-loop). Cellular assays showed that K^768^ did not interfere with other toxin functions, including cell binding, intracellular trafficking, and pore formation. The equivalent K^768^ is conserved among the clostridial neurotoxin family of proteins as a conserved structural motif. The *cis*-loop appears to contribute to LC translocation.

## INTRODUCTION

The clostridial neurotoxins (CNTs), tetanus toxin (TT), and botulinum toxin (BoNT [BT]) serotypes A to G and X (1, 2) are the most toxic proteins for humans, with an estimated 50% lethal dose (LD_50_) of ∼1 ng/kg of body weight for humans (3). In contrast, BTs are used as therapies for numerous human neurological diseases (4, 5). The basis for this extreme potency and such therapeutic applications lies in the neuronal specificity of the CNTs, which bind neuronal receptors and cleave neuron specific soluble N-ethylmaleimide-sensitive factor attachment protein receptors (SNAREs) (6, 7). Clostridia produce CNTs as 150-kDa single-chain proteins, which are cleaved into di-chain proteins that remain linked by an interchain disulfide bond. Di-chain CNT is composed of light chain (LC), which is a Zn^2+^ metalloprotease, and heavy chain (HC), which contains a translocation domain (heavy-chain N-terminal translocation domain [HCN]/HCT) and receptor-binding domain (heavy-chain C-terminal receptor binding domain [HCC]/HCR) (8). CNTs show overall conservation of domain structure but differ in the physical organization of the domains (8–10). CNT interactions with host SNARE substrates and host receptors have been subjected to considerable investigations, but our understanding of LC translocation from the lumen of a vesicle into the cytosol is limited (11). Defining how HCN contributes to LC translocation across host cell membranes will extend our understanding of bacterial toxin action, since diphtheria toxin and other AB toxins may utilize similar translocation mechanisms. Understanding how CNTs translocate LC may enhance the use of BT for human therapy.

Due to regulatory limitations for the production of recombinant, catalytically active tetanus toxin (NIH guidelines for research involving recombinant or synthetic nucleic acid molecules, section III-B-1), a β-lactamase tetanus toxin [TT(RY)] (125,000-fold less toxic than native tetanus toxin [TT]) fusion protein (βlac-TT) was developed as a reporter for a cell-based model to study LC translocation (12). In this model, LC translocation events are detected following cytosolic cleavage of a fluorescence resonance energy transfer (FRET)-based substrate, CCF2, resulting in loss of FRET detected as an unquenched emission of blue-wavelength fluorescence. The βlac-CCF2 reporter system is well established in detecting and characterizing both toxins and effectors (reviewed in reference 13).

**FIG 1:**
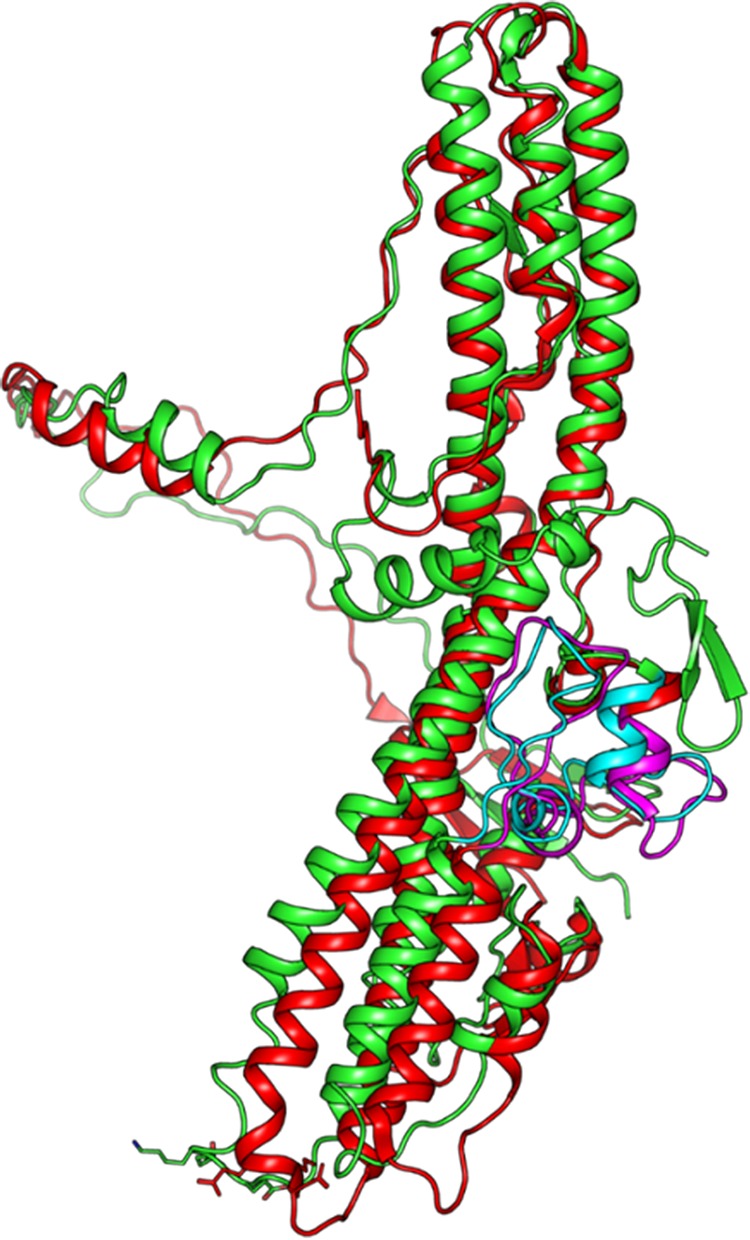
Alignment of HCN of BT and TT. A PyMOL alignment of TT HCN (red) (PDB: 5N0B) was performed on the BT HCN (green) (PDB: 3BTA) with the *trans*-loop at the top and the *cis*-loop at the bottom, showing the BoNT-switch (cyan) and TT equivalent region (magenta) at neutral pH as described by Lam et al. (17).

Among the CNTs, HCN connects to the LC at an interchain disulfide bond (1). The amino acid sequence between the cysteines comprising this disulfide bond is enriched with basic residues and is the site of cleavage by bacterial or host proteases required for CNT activation (14). HCN is composed of seven helices (α12 to α18) (Fig. 1) connected with unstructured loops. HCN has several prominent features. The N-terminal belt is unstructured and comprises ∼80 amino acids (476 to 555), which wraps around the LC and aligns with the α14/15-helices and α16/17-helices within HCN. The α14/15-helices and α16/17-helices are each ∼11 nm in length and are connected to four shorter α-helices by loops (8). The termini of the long α-helices of HCN can also be described relative to their physical distance from the LC-HCN interchain disulfide. While the *trans*-loop region (α18-loop–α18) comprising a CNT-conserved phenylalanine (TT ^838^F equivalent) is located away from the LC-HCN interchain disulfide, the *cis*-loop region (α15-loop–α16) comprising a CNT-conserved lysine (TT ^768^K equivalent) located near the LC-HCN interchain disulfide (1). A long loop termed the membrane penetrating peptide which, in isolation, forms ion conducting channels in lipid membranes aligns with the long kinked α14/15 and α16/17 helices (15, 16). Note that the membrane penetrating peptide is conserved among the CNTs and corresponds to amino acids 641 to 691 of TT (16). Recently, a pleomorphic region in HCN (termed the BoNT switch) may function as a pH-triggered lipid anchor (17). However, how HCN facilitates pore formation and LC delivery remains unknown.

Current models propose that following CNT binding and vesicle entry, acidification triggers HCN insertion into the vesicle membrane to deliver LC into the host cytosol (11, 17–19). LC translocation is necessary for intoxication of host neuron, as premature reduction of the interchain disulfide or inhibition of endosomal acidification reduces neurotoxicity (20). Previous studies on LC translocation identified regions required for pore formation, identifying a minimum pore-forming domain (19), and intoxication as measured by cation conductance through lipid bilayers and substrate cleavage. Heat shock protein 90 (Hsp90) has been shown to contribute in LC translocation (21). Unresolved issues for HCN-mediated LC translocation remain, including what domains/regions are responsible for pore formation and pH-dependent conformational changes, whether pore formation and LC delivery are coupled, and how LC localizes to the pore. The mechanistic role of the interchain disulfide in neurotoxicity is unclear; however, the thioredoxin-thioredoxin reductase (Trx-TrxR) system is involved in the reduction of the interchain disulfide of CNT (22). Conservation of the overall structures of the HCN domain within the crystal structures of BT serotypes and TT (Fig. 1) implicate a common LC translocation mechanism among the CNTs, with the potential for unique properties within individual CNTs based on each CNT’s unique tertiary organization, especially with respect to changes in pH (10).

Here, we used βlac-TT, a discovery-based, live-cell imaging reporter system in cells to map regions of HCN that contribute to LC translocation and identified a helix-loop-helix within HCN (*cis*-loop) required for LC translocation, independently of pore formation.

## RESULTS

### βlac-TT variants in the loop between α15 and α16 (*cis*-loop) within HCN are defective in LC translocation.

**FIG 2:**
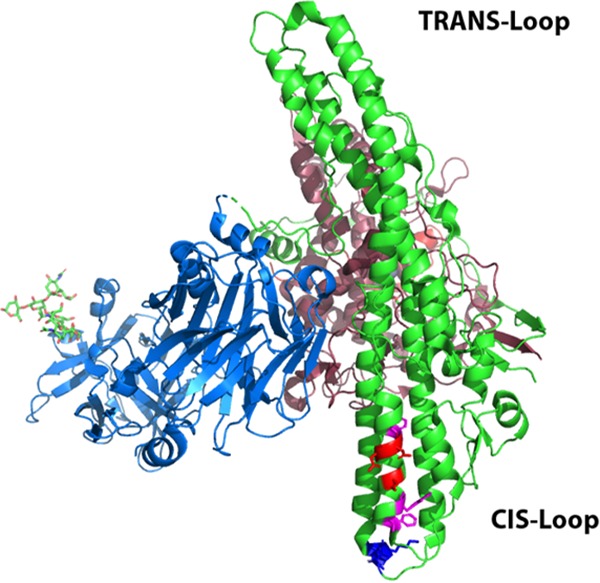
TT-HCN site-directed variants assessed for βlac-LC translocation in intact neurons. The structure of TT (PDB: 5N0B) is shown with *trans*-loop and *cis*-loop designated. Residues were selected for site-directed mutagenesis in α12 (D^618^, D^621^, D^622^) (red) or (F^612^, W^615^, F^623^) (magenta) and a loop between α15 and α16 (^767^DKE^769^) (blue).

βlac-TT was developed as a reporter for a cell-based model of LC translocation by tetanus toxin detected as the cytosolic cleavage of a FRET-based substrate, CCF2 (12). Overall, this assay has proven to be a useful discovery-based system (13). In initial experiments, two conserved regions of the HCN, a hydrophobic region within α12 and a charged loop between α15 and α16 (*cis*-loop) (Fig. 2) were targeted by site-directed mutagenesis to assess potential roles in LC translocation.

**FIG 3:**
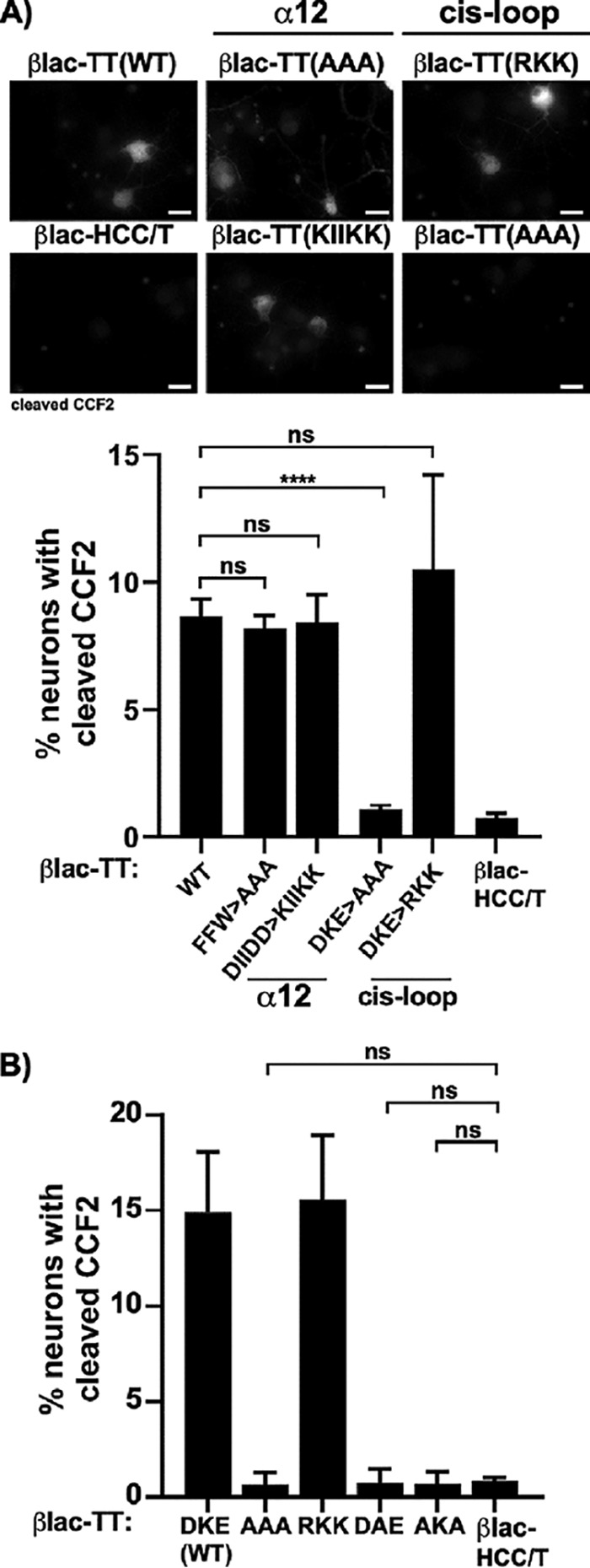
βlac-TT(AAA), a *cis*-loop variant, does not cleave cytosolic β-lactamase substrate CCF2. (A) Primary rat cortical neurons were incubated with 40 nM single-chain βlac-TT (WT [wild type]) and site-directed variants within α12 [βlac-TT(DIIDD>KIIKK) or βlac-TT(^612^A^615^A^623^A)] and the *cis*-loop [βlac-TT(AAA) or βlac-TT(RKK)] for 30 min at 37°C before loading with CCF2 was performed. The top panel shows micrographs of neurons with cleaved CCF2 signal. Bars, 10 μm. The lower panel shows percentages of NeuN^+^ cells containing CCF2 cleavage divided by the total number of NeuN^+^ cells. βlac-HCC/T, which lacks HCN, was used to assess translocation-independent CCF2 cleavage. (B) In addition to βlac-TT (DKE), βlac-TT(AAA), and βlac-TT(RKK), other *cis*-loop variants [K^768^A = βlac-TT(DAE) or D^767^A E^769^A = βlac-TT(AKA)] were assayed for cleavage of cytosolic CCF2 and quantified as described for panel A. Averages of results from three independent replicates are shown with standard errors of the means (SEM). ****, *P* < 0.0001; ns, not significant (Student’s two-tailed *t* test).

Cell imaging showed that mutations within α12 of βlac-TT that targeted a localized group of aspartic acids or three conserved aromatic amino acids translocated βlac [βlac-TT(F^612^A, W^615^A, F^623^A) and βlac-TT(D^618^K, D^621^K, D^622^K)] had a number of βlac translocation events similar to the number seen with βlac-TT (Fig. 3A). In contrast, βlac-TT(^767^AAA^769^), a *cis*-loop mutation, did not cleave cytosolic CCF2 (Fig. 3A). βlac-TT(^767^RKK^769^) mediated numbers of βlac translocation events similar to the number seen with βlac-TT, showing that the acidic residues were not required for βlac translocation. βlac-TT(^767^DAE^769^) and βlac-TT(^767^AKA^769^) did not cleave cytosolic CCF2, indicating that the presence of ^768^K was necessary but not sufficient for reporter translocation (Fig. 3B). Alignment of TT with the seven BT serotypes A to G, BT serotype X, and two recently identified CNT-like proteins, En and Pmp1 (1, 23–25), showed that the *cis*-loop lysine was conserved whereas the acidic amino acids were present but uniquely aligned among the BTs, except BT/C, BT/D, and BT/En, which maintained the DKE sequence (Table 1).

**TABLE 1:** Conservation of the equivalent K^768^ within the *cis*-loop of neurotoxin-like proteins^*a*^

Toxin	Residue-sequence-residue
TT	765-GPDK E-769
BT/A	755-EEEK N-759
BT/B	743-EKEK S-747
BT/C	752-GSDK E-756
BT/D	748-GSDK E-752
BT/E	745-TDEK S-749
BT/F	745-LDEK N-749
BT/G	748-EEDK M-752
BT/X	775-IDDK A-780
BT/En	745-QEDK E-749
Pmp1	716-DNDK L-720
DT	264-SEEK A-268

a BT and TT protein sequences (1, 2, 23–25) were position sequence aligned with multiple-sequence alignment, Clustal Omega (EMBL), or BLAST. The conserved lysine (K) is underlined.

### βlac-TT(^767^AAA^769^) retains protein structure and cell binding function.

**FIG 4:**
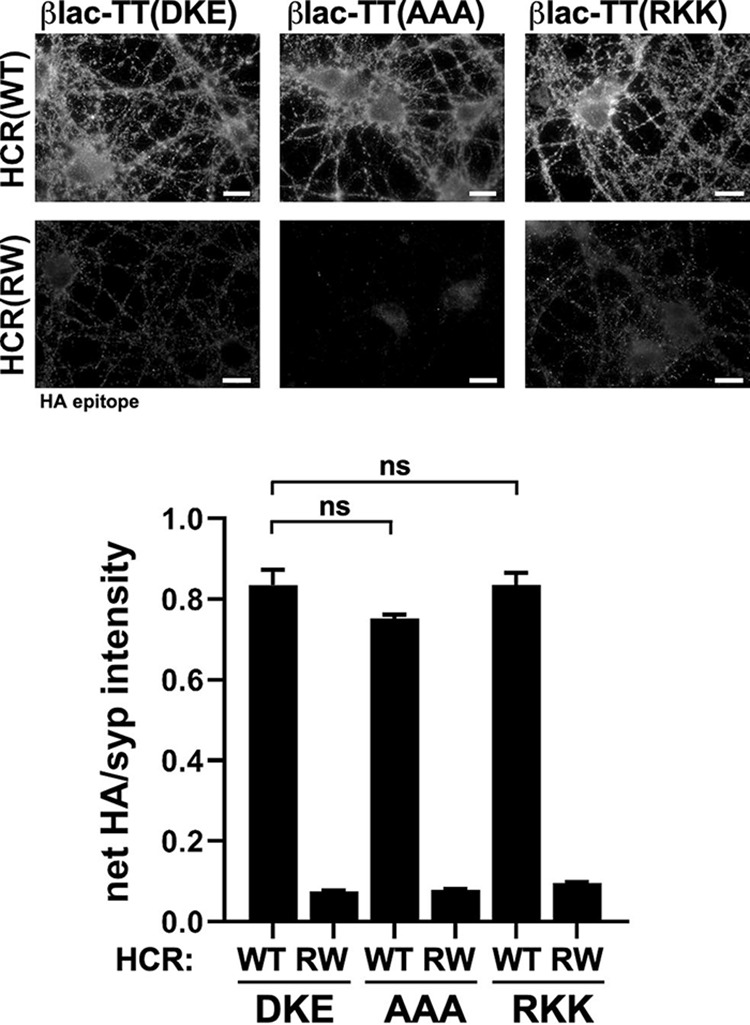
βlac-TT and *cis*-loop variants bind neurons with or without ganglioside association. (Top panel) Neurons were incubated with 40 nM βlac-TT (DKE), βlac-TT(AAA), and βlac-TT(RKK) and βlac-TT^RW^ variants βlac-TT^RW^(AAA) and βlac-TT^RW^(RKK) (“RW” designates mutations with the HCC that inhibit ganglioside binding [R^1226^L, W^1289^A]) for 30 min at 4°C. Fixed cells were probed for C-terminal HA epitope and the synaptic marker synaptophysin (syp). Bars, 10 μm. (Bottom panel) Quantification of net HA normalized to syp. Data representing results from three independent replicates are shown with SEM. ns, not significant (Student’s two-tailed *t* test).

To rule out the possibility that the LC translocation defect was a result of reporter instability or of a lack of disulfide formation, βlac-TT(*cis*-loop) variants were subjected to trypsin digestion. βlac-TT and βlac-TT(^767^AAA^769^) were assembled with the interchain disulfide and yielded similar tryptic cleavage patterns (see Fig. S1 in the supplemental material). In addition, βlac-TT(^767^AAA^769^) (Fig. 4) and various point mutation βlac-TT(^767^DKE^769^) variants bound neurons as efficiently as βlac-TT (Fig. S2), indicating that cell binding by βlac-TT was not disrupted by introduction of *cis*-loop mutations.

10.1128/mSphere.00244-20.1 FIG S1 *cis*-loop variants are stably expressed in E. coli and nick with trypsin as in the case of βlac-TT. (A) A 2-μg volume of purified βlac-TT wild type and *cis*-loop variants was resolved in reducing (βME) buffer by SDS-PAGE. (B) βlac-TT wild type and loop variants were incubated without (-) or with trypsin at the indicated concentrations (1:250 to 1:1,000), boiled, and resolved by SDS-PAGE under oxidizing and reducing conditions. βlac-TT and βlac-TT(AAA) digestions are shown as a representative sample. Download FIG S1, DOCX file, 0.1 MB. Copyright © 2020 Zuverink et al. 2020 Zuverink et al. This content is distributed under the terms of the Creative Commons Attribution 4.0 International license.

10.1128/mSphere.00244-20.2 FIG S2 Binding of TT variants on primary rat cortical neurons. βlac-TT and *cis*-loop variants (40 nM) were incubated with primary rat cortical neurons in precooled low-K^+^ buffer for 30 min on ice as described previously (12). Cells were washed with ice-cold DPBS and processed for immunofluorescence as described in the Fig. 4 legend. Download FIG S2, DOCX file, 0.03 MB. Copyright © 2020 Zuverink et al. 2020 Zuverink et al. This content is distributed under the terms of the Creative Commons Attribution 4.0 International license.

### βlac-TT and *cis*-loop variants traffic to similar vesicles within Neuro-2a cells.

**FIG 5:**
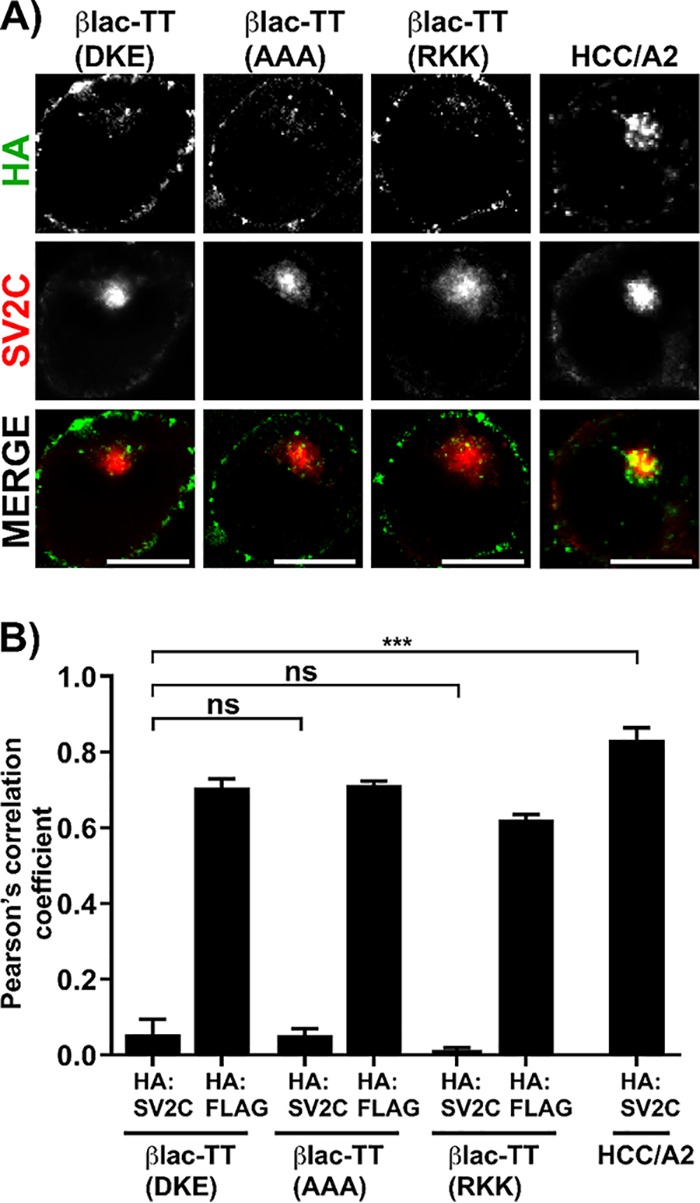
βlac-TT and *cis*-loop variants enter Neuro-2a cells and segregate from synaptic vesicles. (A) Neuro-2a cells enriched with gangliosides were incubated in low-K^+^ buffer with 40 nM βlac-TT (DKE), βlac-TT(AAA), βlac-TT(RKK), and HCC/A2 for 20 min at 37°C. HCC/A2 was used as a control protein that enters Neuro-2a cells and traffics to synaptic vesicle marker protein 2C (SV2C)-positive vesicles (12). Cells were fixed and probed for the N-terminal epitope (FLAG) and the C-terminal epitope (HA) on βlac-TT and *cis*-loop variants and also probed for SV2C. Random single cells containing SV2C signal were deconvolved, and a representative micrograph is shown. Bar, 10 μm. (B) Pearson’s correlation coefficients were determined for N-terminal and C-terminal epitope tags HA:FLAG and HA:SV2C. Averages of results from three independent replicates are shown with SEM. ***, *P* < 0.001; ns, not significant (Student’s two-tailed *t* test).

TT and BT traffic to unique perinuclear vesicles of Neuro-2a cells (12). Like βlac-TT, βlac-TT(^767^AAA^769^) and βlac-TT(^767^RKK^769^) trafficked to the perinuclear vesicles void of synaptic vesicle 2C (SV2C), a synaptic vesicle marker protein, while HCC/A2 trafficked to SV2C-enriched perinuclear vesicles (Fig. 5) (12). HCC/A2, the receptor binding domain of BoNT/A2, was previously shown to traffic to SV2C-enriched perinuclear vesicles (12, 41) Thus, βlac-TT(*cis*-loop) variants were not defective in intracellular trafficking.

### βlac-TT and *cis*-loop variants form similar pores in Neuro-2a cells.

**FIG 6:**
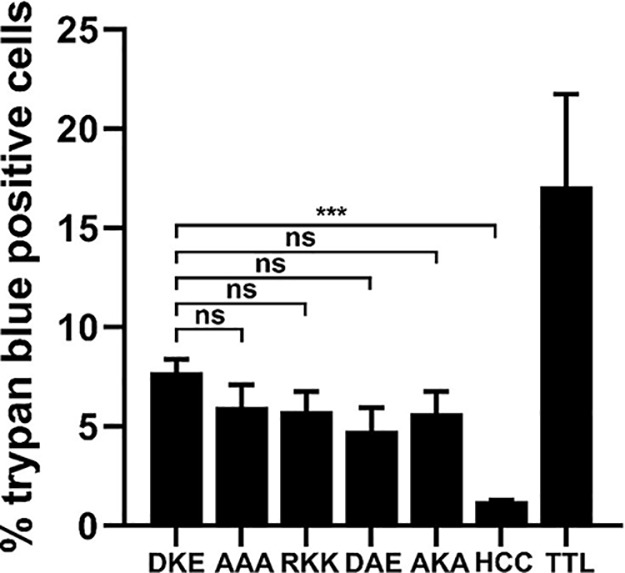
βlac-TT and *cis*-loop variants allow trypan uptake in Neuro-2a cells. (A) βlac-TT(DKE), βlac-TT(AAA), βlac-HCC/T, or tetanolysin (TTL) (80 nM each) was incubated with ganglioside-enriched Neuro-2a cells in low-K^+^ buffer for 20 min at 4°C before pulsing was performed with a prewarmed low-K^+^ reaction mixture buffered with citrate (pH 5.5) for 15 min as previously described (26, 42). Buffer was aspirated and 0.2% trypan blue applied for 1.5 min before fixation. βlac-HCC/T was used to assess translocation-independent trypan uptake, and tetanolysin, a pore-forming toxin, was used as a positive control. Bars, 10 μm. (B) Random fields were obtained, and the number of cells with trypan uptake was expressed as a percentage of the total. Data representing results from three independent replicates are shown with SEM. ***, *P* < 0.001; ns, not significant (Student’s two-tailed *t* test).

Trypan blue diffusion into the cytosol of Neuro-2a cells was used to measure pore formation by cell-bound βlac-TT (26). Tetanolysin was used as a positive control for pore formation. At either 40 nM or 80 nM, βlac-TT or *cis*-loop variants mediated acidification-dependent trypan blue uptake, while βlac-HCC/T, which lacks pore-forming ability, mediated background levels of trypan blue uptake (Fig. 6) (26). This showed that *cis*-loop variants were not defective in pore formation.

### Mutation of *cis*-loop does not alter low-pH- and TrxR-dependent translocation by βlac-TT.

LC translocation by CNTs requires low endosomal pH levels and is inhibited by ionophores or inhibitors of vATPase (27). Previously, βlac-TT translocation was shown to be arrested in the presence of endosomal vATPase inhibitor (12). PropKa analysis (28) of *cis*-loop predicted protonation of acidic residues D^767^ and E^769^ at low pH. Although βlac-TT(^767^AAA^769^) could not be directly assessed, pretreatment of primary neurons with bafilomycin inhibited βlac translocation by βlac-TT(^767^RKK^769^) and βlac-TT (Fig. S3A). More recently, thioredoxin and thioredoxin reductase inhibitors have been shown to be CNT neuroprotective in cells and animals (22). Neurons pretreated with the TrxR inhibitor, auranofin, inhibited βlac-LC translocation by βlac-TT and βlac-TT(^767^RKK^769^) (Fig. S3B). Together, these experiments showed that intrinsic mutations to the *cis*-loop did not alter the canonical pathway of βlac-TT into primary neurons.

10.1128/mSphere.00244-20.3 FIG S3 βlac-TT(RKK) cleavage of cytosolic CCF2 requires low pH and TrxR. Primary rat cortical neurons were preincubated with either 400 nM bafilomycin A1 (A) or 500 nM auranofin (B) for 30 min. Neurobasal media containing the concentrations of inhibitors indicated above with 40 nM single-chain βlac-TT (WT), βlac-TT(AAA), or βlac-TT(RKK) was incubated for 30 min at 37°C before loading with CCF2 was performed. The percentage corresponding to the number of NeuN^+^ cells containing cleaved CCF2 divided by the total number of NeuN^+^ cells is indicated. (B) Three independent replicates are shown with standard errors of the means (SEM). **, *P* < 0.01; *, *P* < 0.05 (Student’s two-tailed *t* test). Download FIG S3, DOCX file, 0.05 MB. Copyright © 2020 Zuverink et al. 2020 Zuverink et al. This content is distributed under the terms of the Creative Commons Attribution 4.0 International license.

### Molecular simulations detected a polarity in HCN *cis*-loop orientation with the cell membrane.

**FIG 7:**
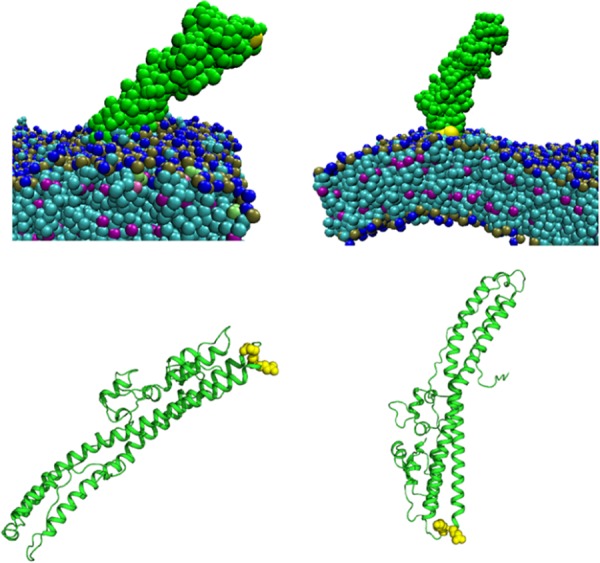
Molecular models of the *cis*-loop and HCN coarse-grain simulations of beltless HCN (green, with ^767^DKE^769^ in yellow) with a lipid membrane (blue, teal, and magenta). A representative molecular dynamics model of beltless HCN (residues 564 to 870) with mutations to mimic protonation of acidic residues D621N, D634N, D638N, E675Q, E679Q, E717Q, and D767N is shown. Nine of 10 simulations had long-term interactions with the membrane and in eight simulations (left) the *trans*-loop of the HCN was oriented toward the membrane, while once (right) the *cis*-loop of the HCN oriented toward the membrane. The upper HCN images include the membrane and the lower HCN images exclude the membrane.

CNT-HCNs comprise a *trans*-loop (α18-loop–α18 (^838^F in TT), distanced from the LC-HCN interchain disulfide, and a *cis*-loop (α15-loop–α16) (^767^DKE^769^ in TT) near the LC-HCN interchain disulfide (1). Protein model alignments showed TT(RY) (PDB: 5N0B) contained an analogous BoNT-switch like BT/A (PDB: 6DKK) (Fig. 1), implicating a similar mechanism of pH-dependent membrane insertion among the CNTs. Molecular dynamics simulations of beltless HCN with a lipid membrane (29) showed the preferred association, i.e., 8 of 9 simulations that yielded a membrane-bound HCN, of the HCN *trans*-loop with the cell membrane, with the HCN *cis*-loop angled off the cell membrane (Fig. 7). After the initial association with the membrane, HCN remained bound and the HCN *trans*-loop was buried in the membrane. The surface orientation of the *cis*-loop in these simulations is consistent with the *cis*-loop functioning independently of pore function.

## DISCUSSION

LC translocation is the least understood step in CNT intoxication. This study characterized the role of conserved regions of the HCN required for LC translocation in TT, assuming that the mechanism of LC translocation is conserved among CNTs. In neurons, deletion of conserved loops and short helices of HCN in C-terminal regions relative to α12 reduced LC translocation efficiency, while deletion of N-terminal loop regions relative to α12 was dispensable for LC translocation (unpublished data). In addition, a short stretch of amino acids, ^767^DKE^769^, within the α-helix15-loop–α-helix16 junction (*cis*-loop) was required for LC translocation but not intracellular trafficking or pore formation. Consistent with a role in orientating the unfolded LC for translocation were molecular simulations that located the *cis*-loop on the membrane surface (Fig. 7). These studies identified the *cis*-loop as participating in LC translocation, independently of pore formation. Directed mutagenesis of βlac-TT identified a charged loop within the HCN of TT, the *cis*-loop comprising ^767^DKE^769^ within α-helix15-loop–α-helix16 that mediated βlac-LC translocation. The aliphatic substitution of βlac-TT(^767^AAA^769^) also failed to translocate βlac-LC. ^768^K was the only conserved amino acid within the *cis*-loop of the respective CNTs, supporting the idea of the *cis*-loop being a structural motif.

The *cis*-loop structure is conserved in all BT serotypes, and the DKE sequence was homologous in BT/C and BT/D, but varied within the *cis*-loop of the other BT serotypes (Table 1). Thus, the *cis*-loop appears to be a structural motif rather than a sequence-based motif. Alignments with diphtheria toxin identified a candidate *cis*-loop, ^239^SEEKA^243^, within α-TH5–α-TH6, which is N terminal to the membrane-penetrating α-TH8–α-TH9 (PDB: 1SGK). Since α-TH5–α-TH6 has some membrane interaction properties, the *cis*-loop could contribute in membrane stability (30). The TT-*cis*-loop was similar to the *cis*-loop of BT/En and homologous to the *cis*-loop of an *Anopheles* mosquito protein (25). Thus, the *cis*-loop is a feature shared among bacterial toxins that possess an HCN-like LC translocation function.

**FIG 8:**
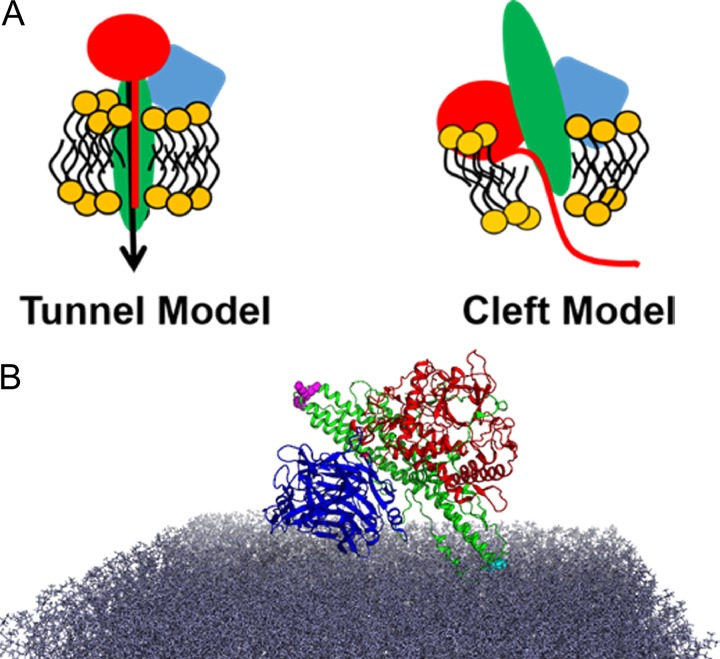
Tunnel model and cleft model for LC translocation. (A) Schematics of two earlier proposed models (6, 11, 33) for HCN-mediated delivery of LC across a vesicle membrane. In the tunnel model, unfolded peptide translocates through a proteinaceous pore. In the cleft model, HCN deforms the membrane and the LC partially unfolds, ratcheting through the deformed lipid membrane, where a hydrophobic core of HCN contacts lipids and hydrophilic residues of LC contact the HCN. Supporting the cleft model are findings showing that BT in lipid membranes can conduct ions but not larger molecules (33). (B) LC and HCC were modeled into the simulation of membrane-bound beltless HCN with a lipid membrane (see Fig. 7, left) and are shown as follows: red, LC; green, HCN; blue, HCC; gray, lipid membrane. Residues are highlighted as follows: magenta, *cis*-loop ^767^DKE^769^; cyan, *trans*-loop ^838^F.

CNT-mediated LC translocation has been predominantly studied using electrophysiological measurements of cation flow across lipid membranes, liposomal release assays, or analysis of substrate cleavage in neuronal cells. These studies used full-length CNT or isolated domains and aided our appreciation that low pH, proteolysis, and an intact disulfide (12, 20, 31, 32) were involved in LC translocation. Two models of LC translocation for BT included the tunnel model and the cleft model (Fig. 8) (6, 11, 33). In the cleft model, HCN deforms the membrane and the LC partially unfolds, ratcheting through the deformed lipid membrane where a hydrophobic core of HCN contacts lipids and hydrophilic residues of LC contact the HCN. Supporting this model are the findings that the protein toxin channels in lipid membranes can conduct ions but not larger molecules (33). Both translocation models require energy to drive the processes of pore formation and LC translocation. Protein toxins, such as diphtheria toxin and ricin toxin, require proton gradients and membrane potential to drive membrane insertion (34). The energy requirement for CNT LC translocation has not been resolved but may be due to Brownian motion (35). Brownian motion is a passive diffusion of polypeptides through the translocon, which results from protonation and neutralization of acidic residues that then deprotonate upon cytosolic delivery. Deprotonation of acidic residues increases repulsion between the translocated polypeptide and the acidic phospholipids, driving LC translocation (36, 37). Independently of the channel, LC translocation also involves cytosolic host factors, such as Hsps, as chaperones to facilitate delivery that is ATP dependent (21, 36, 38). The cleft model requires acidic residue neutralization or shielding of charged residues, such as pairing basic catalytic domain residues with acidic phospholipid headgroups or anions, to be energetically favorable (33). The *cis*-loop function is compatible with either the tunnel model or the cleft model for LC translocation. The *cis*-loop may function in LC translocation directly by orientating the LC for delivery into the pore; mammalian J proteins have this type of structure-function (39), since the *cis*-loop is located adjacent to the interchain disulfide (LC-HCN) that was previously shown to be needed for LC translocation (12, 40). Alternatively, the *cis*-loop may contribute to the pH-dependent conformational changes, since each CNT *cis*-loop possesses, in addition to the conserved K^768^, at least one adjacent acidic amino acid that may change charge with pH (Table 1).

Continued assessment of α-helix15-loop–α-helix16 (*cis*-loop) and amino acids that define the structural components of the *cis*-loop may provide additional details for LC translocation.

## MATERIALS AND METHODS

### Tetanus toxin reporter expression.

βlac-TT(RY) and βlac-HCC/T were previously engineered (12, 41). DNA encoding atoxic βlac-TT(RY) was used as a template for site-directed mutagenesis of HCN. Site-directed mutagenesis primers designed for use an Agilent QuikChange II kit or a New England Biolabs Q5 SDM kit were used to engineer βlac-TT *cis*-loop (^767^DKE^769^ > ^767^AAA^769^ and ^767^DKE^769^ > ^767^RKK^769^) per kit instructions before transformation into Escherichia coli TG1. Primers for site-directed mutagenesis of the other βlac-TT *cis*-loop variants [residues K^768^A (DKE > DAE) and D^767^A and E^769^A (DKE > AKA)] and βlac-TT α12 mutations [residues D^618^K, D^621^K, and D^622^K (DIIDD > KIIKK) and F^612^A, W^615^A, and F^623^A (FWF > AAA)] were designed using Agilent or NEB Web-based tools. Briefly, templates and primers were mixed and amplified per the specifications in the Q5 kit instructions (New England Biolabs). Amplified product was incubated with KLD (kinase, ligase, and DpnI) before transformation of the entire reaction into E. coli TG1.

βlac-TT and *cis*-loop variants were transformed into E. coli BL21(DE3) for expression and purified as previously described (12, 41). Clarified soluble fractions were purified by tandem gravity-chromatography using nickel-nitrilotriacetic acid (Ni-NTA) agarose (Qiagen) followed by Strep-Tactin high-capacity resin (IGA Life Sciences), concentrated using a 0.5-ml Amicon centrifugal 10K-cutoff filter (EMD Millipore), and stored at 4°C.

### Trypsin sensitivity and βlac activity of TT variants.

βlac-TT and *cis*-loop variants were incubated with trypsin (Sigma-Aldrich) at 1:1,000 to 1:250 (wt/wt) trypsin to variant in 20 mM sodium phosphate (pH 7.9)–50 mM NaCl for 1 h at 37°C to assess stability. Samples were inhibited every 10 min with 5 molar excess soybean trypsin inhibitor (Sigma-Aldrich). Proteins were subjected to 8% acrylamide SDS-PAGE (with beta-mercaptoethanol [βME]) and stained with Coomassie blue. βlac activity was assayed using fluorocillin green microplate assay (Thermo Fisher) (12).

### Cell culture protocols.

Neuro-2a cells (ATCC CCL-131), from a Mus musculus neuroblastoma, were cultured as described previously (12) with the exception that coverslips were coated with poly-d-lysine (Sigma-Aldrich) followed by assay 1 day after plating at ∼70% confluence. E18 rat cortices from Sprague Dawley rats (BrainBits, LLC) were triturated to single cells as described by the supplier and plated in NBActiv4 (BrainBits, LLC) (45,000 cells/well) on glass-bottom total internal reflection (TIRF) plates (MatTek). TIRF plates were precoated with 20 μg/ml poly-d-lysine (Sigma-Aldrich) overnight, followed by 3 μg/ml mouse laminin for 3 h, and equilibrated with neurobasal medium for 30 min before plating cells in NBActiv4. Neurons were cultured for 7 to 12 days with a half-fresh media change, using NBActiv1 (BrainBits, LLC) on days 4 and 7 postplating.

### Trypan blue uptake assay (pore formation) of βlac-TT variants in Neuro-2a cells.

Trypan blue uptake was performed as previously described (26). Briefly, cells were plated as described above and loaded with 10 μg/well of GT1b. Cells were washed with cooled low-K^+^ buffer (15 mM HEPES, 145 mM NaCl, 5.6 mM KCl, 2.2 mM CaCl_2_, 0.5 mM MgCl_2_, pH 7.4) and incubated on ice for 10 min. βlac-TT or scanning deletion variants or *cis*-loop variants (40 or 80 nM) were suspended in cooled low-K^+^ buffer and incubated with cells for 20 min on ice, buffer was replaced with prewarmed low-K^+^ buffer to reach pH 5.5 with 5 mM sodium citrate for 15 min, and the reaction mixture was incubated at 37°C (42). Cells were aspirated, 300 μl of 0.2% trypan blue was added for 1.5 min, and the reaction mixture was aspirated and fixed with 4% paraformaldehyde (PFA). The clostridial pore-forming toxin tetanolysin (List Biological Laboratories, CA) was used as a positive control for pore formation. Several negative controls, including ganglioside-loaded cells incubated at pH 7 or pH 5.5 with βlac-HCC/T as a measure of translocation-independent trypan blue uptake, were used to assess pore formation-independent trypan blue uptake. βlac-TT was incubated at pH 7 to confirm pH-dependent trypan uptake.

### Entry and intracellular trafficking of βlac-TT variants into Neuro-2a cells.

Plated Neuro-2a cells were loaded with 10 μg/well of sonicated ganglioside GT1b (Matreya) in minimal essential medium (MEM) containing 0.5% fetal bovine serum (FBS) for 4 h at 37°C. Cells were washed with prewarmed Dulbecco’s phosphate-buffered saline (DPBS) and incubated with 40 nM βlac-TT or variants in low-K^+^ buffer for 20 min, at which time cells were washed and processed for immunofluorescence (described below). Cells were located as SV2C-positive stained cells, and Z-stacks were taken at 0.4-μm steps followed by cropping individual cells and blind deconvolution to estimate the point spread function (PSF) with 15 iterations.

### Binding of TT variants on primary rat cortical neurons.

βlac-TT and variants were incubated (10 or 40 nM) with precooled primary rat cortical neurons in low-K^+^ buffer for 30 min on ice as described previously (12). Cells were washed with ice-cold DPBS and processed for immunofluorescence.

### LC translocation assay in primary rat cortical neurons.

Rat cortical neurons were incubated in equilibrated neurobasal media containing (10 or 40 nM) single-chain βlac-TT or variants for 30 min at 37°C. Neurons were washed with Hanks’ balanced salt solution lacking Ca^2+^ and Mg^2+^ (HBSS^−/−^) (Life Technologies) and cooled to room temperature (RT) for 10 min followed by loading of media with 2 μM CCF2-AM (Life Technologies) and 1 mM probenecid (Life Technologies), an anion transport inhibitor, in HBSS^−/−^ for 30 min. Neurons were washed and processed for immunofluorescence. The inhibitor-treated translocation assay was performed as described above, with the following preincubation modifications. Bafilomycin A1 (400 nM), a vesicular ATPase inhibitor (Sigma-Aldrich), was preincubated with neurons in neurobasal media for 30 min at 37°C before aspiration and application of 40 nM single-chain βlac-TT in neurobasal media containing inhibitor at the indicated concentration. Then, auranofin (500 nM), a thioredoxin reductase inhibitor (Sigma), was preincubated with neurons for 30 min at 37°C before its removal and addition of 40 nM single-chain βlac-TT in neurobasal media containing inhibitor at the indicated concentration. After being maintained 30 min with βlac-TT at 37°C, cells were washed and loaded with CCF2 as described above followed by fixation.

### Immunofluorescence assays.

Primary cortical neurons and Neuro-2a cells were fixed and permeabilized (12). Primary neuron cultures were incubated with primary antibody overnight at 4°C with 1:1,000 rabbit anti-neuron-specific nuclear protein (NeuN) (EMD Millipore), 1:12,000 mouse anti-FLAG (Sigma-Aldrich), or 1:2,000 guinea pig anti-synaptophysin. Neuro-2a cells were also incubated with 1:2,000 rat anti-hemagglutinin (HA; Roche) or with 1:2,000 rabbit anti-synaptic vesicle glycoprotein 2C (SV2C) (Synaptic Systems) or in buffer without primary antibody (12). For Neuro-2a cells, coverslips were mounted and cured in ProLong Gold (Life Technologies) on glass slides. For neurons, Citifluor AF-3 antifade reagent (Electron Microscopy Sciences) was added prior to analysis. Micrographs were collected using a Nikon inverted microscope by epifluorescence using a CFI Plan Apo 60× oil objective (numeric aperture, 1.49) and 20× objective (numeric aperture, 0.45) and a Photometrics CoolSnap HQ2 camera. For primary neuron translocation quantitation, micrographs were collected at 20×. For primary neuron binding and Neuro-2a entry, respectively, 60× micrographs and Z-stacks were taken (12).

### Data analysis.

Secondary Alexa-conjugated antibody-only controls were used to subtract autofluorescence and background fluorescence emitted by cells, yielding the net average intensity of fluorescence. FLAG or HA epitopes were normalized to a cellular marker such as synaptophysin. For protein binding and inhibitor-treated neurons, neurons were localized with guinea pig anti-synaptophysin and rabbit anti-NeuN, respectively. For the measurement of LC translocation following inhibitor treatment, clusters of 3 to 6 neurons were located as NeuN-positive cells with a 60× objective and assessed for cleaved CCF2 substrate above the no-protein control. To compare the translocation efficiencies of *cis*-loop variants, micrographs were acquired at ×20 magnification and neurons containing cleaved CCF2 were scored as positive/negative for βlac-LC translocation. At least 10 fields (containing 20 to 30 neurons) were analyzed per experiment, and at least 3 independent experiments were performed. For entry of *cis*-loop variants into Neuro-2a cells, Z-stacks were acquired and deconvolved. Pearson’s correlation coefficient was obtained using Nikon AR analysis software as described previously (12).

### Molecular simulations of the beltless TT(HCN).

Gangliosides were removed from the crystal structure file of TT (PDB: 5N0B), and the structure was truncated to residues 564 to 870 [TT(HCN)], using PyMol. A coarse-grain structure with an elastic network was generated using the martinize.py script (version 2.2) with secondary-structure assignment provided by the Define Secondary Structure of Proteins (DSSP) program (version 2.0.4). Lipid and amino acid parameters were taken from Martini Lipidome and Martini (version 2.2). Simulations were performed as follows. The force field used for generating the coarse-grain structure was martini22, with an elastic bond force constant (-ef) of 500, an elastic bond upper distance bound (-eu) of 0.9, and an elastic bond lower bound (-el) of 0.5. The lipid bilayer system was generated using the insane.py script with a 20-, 20-, 30-nm box (xyz) with 48% phosphatidylcholine, 31% phosphatidylethanolamine, 9% phosphatidylserine, 5% sphingomyelin, and 3% phosphatidylinositol to mimic the mixtures of lipids found in neuronal vesicles. Using GROMACS 2018 (released 10 January 2018), the energy of the system was minimized for 2,000 steps with a 0.02-ps step size (40 ps) using the steep integrator. The system then underwent temperature equilibration from 0 to 300 K using v-rescale temperature coupling for 10,000 steps with a 0.01-ps step size (100 ps) and the md (leap-frog) integrator. Then, the system was equilibrated to 1 bar using the Berendsen barostat with semi-isotropic pressure coupling for 50,000 steps with a 0.01-ps step size (500ps) and the md (leap-frog) integrator. The system was finally equilibrated to 1 bar using the Parrinello-Rahman barostat with semi-isotropic pressure coupling for 50,000 steps with a 0.01-ps step size (500 ps) and the md (leap-frog) integrator. Simulations were carried out on MCW Research Computing Cluster Tesla using Tesla K40 and K80 graphics processing units (GPUs). Trajectories were converted and analyzed using VMD (version 1.9.3).

### Statistics.

Student’s unpaired, two-tailed *t* test was utilized to determine if two data sets were significantly different where appropriate.
