# Supplementary figures and images for: Tetanus Toxin cis-Loop Contributes to Light-Chain Translocation
Source: mSphere. 2020 May 6;5(3):e00244-20. doi: 10.1128/mSphere.00244-20 (PMC7203457; doi:10.1128/mSphere.00244-20)

**
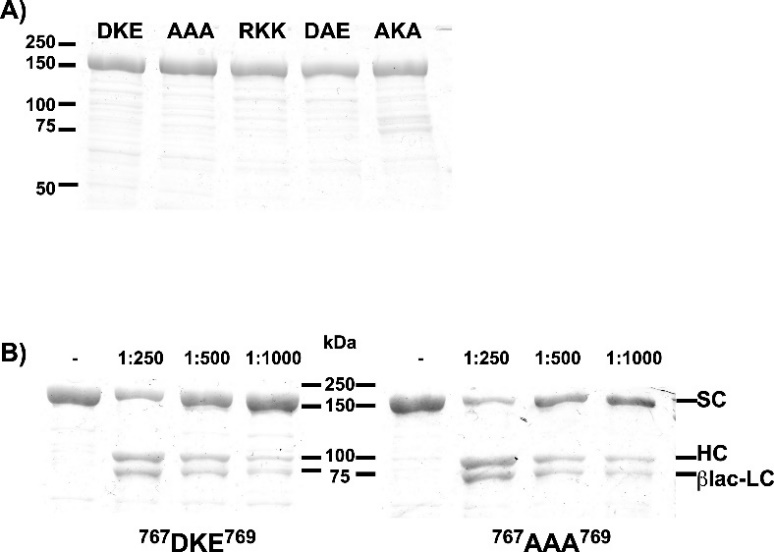
**

Supplement: FIG S1 [file mSphere.00244-20-sf001.docx]

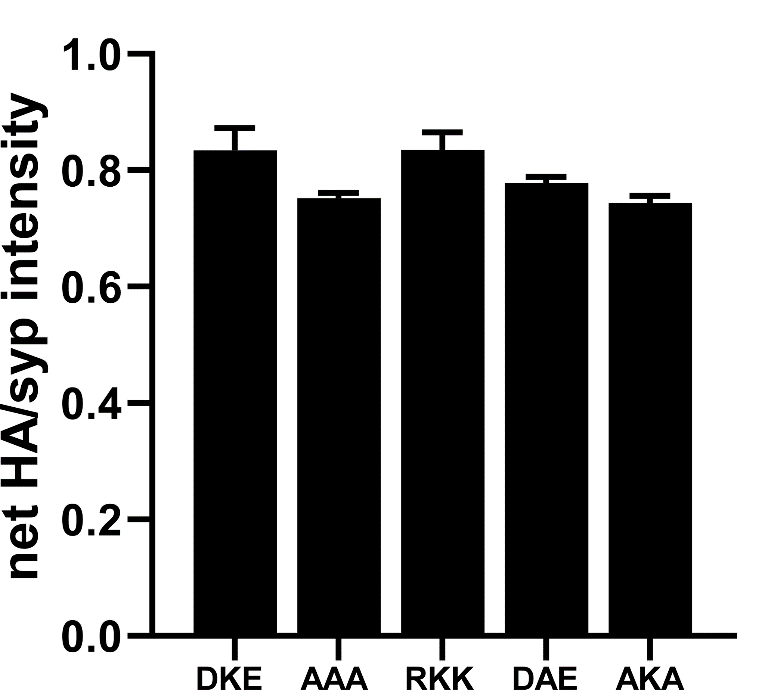

Supplement: FIG S2 [file mSphere.00244-20-sf002.docx]
